# An economic valuation of federal and private grazing land ecosystem services supported by beef cattle ranching in the United States

**DOI:** 10.1093/tas/txab054

**Published:** 2021-05-04

**Authors:** Anna T Maher, Nicolas E Quintana Ashwell, Kristie A Maczko, David T Taylor, John A Tanaka, Matt C Reeves

**Affiliations:** 1 Ecosystem Science and Management, University of Wyoming, Laramie, WY 82071, USA; 2 National Center for Alluvial Aquifer Research, Mississippi State University, Leland, MS 38756, USA; 3 Agricultural and Applied Economics, University of Wyoming, Laramie, WY 82071, USA; 4 USDA Forest Service, Rocky Mountain Research Station, 800 E. Beckwith Ave, Missoula, MT, USA

**Keywords:** beef cattle, economic valuation, ecosystem services, federal, rancher, rangelands

## Abstract

Beef cattle ranching and farming is a major agricultural industry in the United States that manages an estimated 147 million ha of private land and uses approximately 92% of forage authorized for grazing on federal rangelands. Rangelands, as working landscapes, sustain beef cattle ranching while providing habitat for wildlife, recreation, and open space amenities, as well as spiritual and cultural values that define a way of life. Historically, discussions regarding the economics of beef cattle ranching have focused primarily on the value of beef production but have more recently expanded to consider related ecosystem services. A systematic search of peer-reviewed literature published between 1998 and 2018 found 154 articles that considered ecosystem services from rangelands/grasslands. Of these, only two articles (1%) provided an in-depth economic valuation (monetary measure) of ecosystem services in the United States. To fill this knowledge gap, we primarily used publicly available data to conduct an economic valuation of major ecosystem services associated with beef cattle production in the United States at both the national and state levels. We find that over 186 million ha were actively grazed by beef cattle ranches and farms in the United States in 2017. We estimate the economic value of this land use to be $17.5 billion for wildlife recreation, $3.8 billion for forage production, and $3.2 billion for other ecosystem services related to the conservation of biodiversity—a combined total of $24.5 billion. Ecosystem services from federal rangelands in 16 western states accounted for 35% of the total value. Ecosystem services per beef cow and per kilogram of retail beef were estimated to be $1,043.35 and $2.74, respectively. More studies like these are needed to inform decision-makers at the industry, land management, and federal levels to ensure that the conservation, improvement, and restoration of these ecosystem services are considered in future management and research efforts.

## INTRODUCTION

Beef cattle ranching and farming is a major agricultural industry in the United States. The 2017 Census of Agriculture reported that 641,500 beef cattle ranches and farms generated $34.7 billion of annual gross revenue and managed 146.7 million ha of private land (USDA, 2019). This industry utilized 68% (111.0 million ha) of land identified as private permanent rangeland/pastureland in the 2017 Census of Agriculture (USDA, 2019) and an estimated 92% of the forage authorized for grazing on federal rangelands in the United States (USFS, 2017; BLM, 2019). The rangelands that support beef cattle ranching are also thought to offer commodity, amenity, and spiritual values (Maczko et al., 2016), support a way of life (Gentner and Tanaka, 2002), and provide habitat for wildlife while contributing to recreation and open space amenities (Maczko and Hidinger, 2008).

Although discussions regarding the economics of beef cattle ranching have primarily focused on the value of beef production, this is one of a myriad of benefits that humans derive from rangelands. The Millennium Ecosystem Assessment identified four categories of ecosystem services (benefits from ecosystems to humans): 1) provisioning, for instance production of food and water; 2) regulating, which broadly describes the control of climate and disease; 3) supporting, such as nutrient cycles and crop pollination; and 4) cultural, including spiritual, cultural, and recreational benefits (MEA, 2005). Maczko and Hidinger (2008) used a traditional market, non-market tangible, and intangible classification system of ecosystem goods and services specifically for rangelands. Research has also indicated that ranchers care about more than provisioning and also manage for a number of other cultural, regulating, and supporting services (Gentner and Tanaka, 2002; Lind, 2015; Collins, 2019; York et al., 2019). Therefore, following an approach similar to Rashford et al. (2013) and Taylor et al. (2019), we use the term “beef cattle ranching-based ecosystems services” to refer to values beyond the category of provisioning services.

Studies quantifying the economic value of ecosystem services from rangelands are few. A search of peer-reviewed literature published between 1998 and 2018 found 154 articles that considered ecosystem services from grasslands and rangelands. Of these, only two articles provided an in-depth economic valuation (monetary measure) of these ecosystem services in the United States. Rashford et al. (2013) considered ecosystem service values associated with grazing lands for 17 U.S. states in the West, and USDA-NRCS (2010), another study, provided a benefit–cost analysis of the Grassland Reserve Program. More recently, Taylor et al. (2019) provided a valuation of beef cattle ranching-based ecosystems services as associated with private grazing lands but did not include federal grazing lands.

Despite the limited number of valuation studies, some of the literature suggests significant value associated with the flow of ecosystem services from pasture and rangelands to society through beef cattle ranching. Pogue et al. (2018) reviewed the literature and concluded that beef cattle ranching in Canada’s prairie provinces had a positive influence on biodiversity, habitat maintenance, cultural heritage, and recreation/tourism. Havstad et al. (2007) identified, but did not quantify, existing and diminished or diminishing ecosystem goods and services from rangelands in the United States. Costanza et al. (2014) considered grassland ecosystem service values at a global scale. Fox et al. (2009) and Maczko et al. (2011) developed a qualitative framework to assess rangeland ecosystem goods and services for the purpose of identifying and weighing potential alternative income streams for ranchers.

The existence of federal (CRP, 2020; NRCS, 2020; SGI, 2020) and nonprofit (TNC, 2013) programs that support working rangelands are also evidence of the societal value in beef cattle ranching. These conservation programs help address existing concerns regarding past and future rangeland conversion to other land uses and declining rangeland health. Expanding crop production (Hongli et al., 2013; Haggerty et al., 2018; WWF, 2018), population growth (Brunson and Huntsinger, 2008; Brunson et al., 2016; Farley et al., 2017; Reeves et al., 2018), and cheatgrass (*Bromus tectorum* L.) invasion (Brooks et al., 2004; Chambers et al., 2014; Pellant, 2018, Williamson et al., 2020) are just a few examples of the land use and management challenges involved in conserving the flow of ecosystem services from rangelands. The losses or diminishment of these ecosystem services may be irreversible or difficult to recover (Salles, 2011; Bestelmeyer et al., 2015; Pellant et al., 2018; WWF, 2018).

The purpose of this study is to address informational gaps that exist regarding the sustainability of beef cattle production through conducting a formal and extensive valuation of major U.S. beef cattle ranching-based ecosystem services at the state and national level. Although the value of ecosystem services is difficult to quantify (Torell et al., 2014c; Brown and MacLeod, 2018), such valuation can provide vital information in land management decision-analysis and in assessing the cost to society from changes in land use. This valuation is also useful for identifying alternative income sources for ranchers and to provide information for the development of ecosystem services markets (Maczko et al., 2011). Building on the methods used in Taylor et al. (2019) and Rashford et al. (2013), this study estimates the economic value of ecosystem services from both private and federal lands for three major ecosystem services associated with beef cattle production: 1) wildlife-related recreation, 2) forage production, and 3) other ecosystem services.

## MATERIALS AND METHODS

As in Rashford et al. (2013) and Taylor et al. (2019), this study used publicly available data to estimate the economic value of major benefits from beef cattle ranching related ecosystem services in the lower 48 U.S. states (Alaska and Hawaii were not included in this analysis due to data limitations). Following Costanza et al. (2014), it is assumed ecosystem services are constant across space, an approach thought to be appropriate for assessing land use change scenarios over larger areas. Dollar amounts are indexed to the year of the most recent Census of Agriculture (2017) using the Consumer Price Index.

The per hectare value of three categories of ecosystem services were estimated: 1) wildlife-related recreation, 2) forage production, and 3) other ecosystem services. The aggregate of the three categories is also presented on a per hectare basis. Private land and federal land values were found separately. Total ecosystem services value on private rangelands was found by multiplying the aggregate value per hectare by the number of hectares of private rangeland and pasture under beef cattle production in each area as reported by the 2017 Census of Agriculture under the North American Classification System (**NAICS**) code 112111 for “Beef Cattle Ranching and Farming” (USDA, 2019). Similarly, the aggregate per hectare value for federal land was multiplied by the estimated number of hectares grazed by cattle. Details about this estimation process can be found in the Materials and Methods section under “Forage Production.”

These total ecosystem service values estimated separately for private and federal land were then summed to obtain the combined ecosystem services value associated with beef cattle ranching from both private and federal lands in each geographic area. To calculate ecosystem services value per beef cow, this summed federal and private value was divided by the number of beef cows reported in the 2017 Census of Agriculture (USDA, 2019) under NAICS code 112111. To calculate the ecosystem services value per pound of beef, the value per beef cow was then divided by the number of kilograms of retail beef per beef cow from the Livestock Marketing Information Center (LMIC, 2018). Detailed descriptions of the per hectare value estimation methods for each of the three types of ecosystem service categories follow.

### Recreation

Wildlife recreation values were found by combining U.S. Fish and Wildlife Service (**USFWS**) estimates of the number of recreation days (hunting, freshwater fishing, excluding Great Lakes fishing, and wildlife watching) per year (USFWS, 2014), with USFWS estimates of net economic values for wildlife-related recreation per day (USFWS, 2016). Per hectare values were calculated by dividing the total wildlife recreation value in each state by the number of hectares in non-metro and nonurban land (Headwater Economics, 2018).

### Forage Production

Rangeland/pasture forage is an input to livestock production, the value of which depends upon its contribution to the final market value of livestock (Rashford et al., 2013). In a perfectly competitive market, grazers would be willing to pay the amount that this forage contributes to the value of the final good (kilograms of beef in this case). This study therefore approximates forage production values on private lands by using United States Department of Agriculture National Agricultural Statistic Service (USDA NASS) pasture rental rate data (NASS, 2017).

Estimating the value of forage per hectare on federal land required information about the spatial location (state level) and quantity of federal forage as well as the dollar value per Animal Unit Month (**AUM**). The number of grazing hectares utilized by beef cattle on federal land was found by employing several data sources. First, grazing allotment boundaries were determined from publicly available geographical information system (**GIS**) data (BLM, 2020; USFS, 2020). Second, vegetated area in these active grazing allotments was found from Landscape Fire and Resource Management Planning Tools (LANDFIRE at https://www.landfire.gov/) Existing Vegetation Type (EVT) (Comer et al., 2003; Rollins, 2009). Land cover classes such as water, urban, agricultural, pasture, and barren were excluded from this data set to represent only natural vegetation (excluding pasture classes if any occurred in the allotment). Third, only federal lands were included in the land cover classes as identified using the Protected Areas Database of the United States (**PADUS**) (CBI, 2021). Only lands managed by the BLM and USFS were retained for analysis from this spatially explicit database. The grazing allotments and PADUS data that are natively offered in vector format were converted to raster data format at 30-m spatial resolution to match the extent and pixel size of the EVT data. A spatial subset was created by spatially intersecting this data with active grazing allotments data. Finally, the state area found through the GIS analysis was multiplied by the percentage of AUMs grazed by cattle in each state (Table 1) to provide an estimate of federal area specifically grazed by cattle rather than other forms of livestock. Due to data limitations, only BLM and FS owned land was considered for this study. Grazing also occurs on other federally owned lands such as USFWS land, but the majority of cattle grazing on federal land in the United States are on BLM and FS land.

**Table 1.** Animal Unit Months (AUMs) and percent of total AUMs grazed by cattle on Bureau of Land Management (BLM) and U.S. Forest Service (USFS) land (BLM, 2019; USFS, 2017) for the 16 states governed by the federal grazing fee

	BLM and USFS AUMs		
U.S. state	Total	Cattle	Percent cattle
Arizona	1,412,541	1,373,085	97
California	553,997	513,385	93
Colorado	1,131,718	976,434	86
Idaho	1,596,902	1,416,235	89
Kansas	25,917	25,917	100
Montana	1,608,147	1,563,895	97
Nebraska	106,052	106,042	100
Nevada	1,512,008	1,371,660	91
New Mexico	2,182,165	2,090,679	96
North Dakota	547,494	547,255	100
Oklahoma	14,126	14,126	100
Oregon	1,197,710	1,168,894	98
South Dakota	476,508	473,573	99
Utah	1,460,153	1,149,984	79
Washington	104,825	100,992	96
Wyoming	1,803,255	1,600,202	89
Total	15,733,517	14,492,358	92

Valuing forage from federal grazing lands is not a straightforward process though many researchers have attempted to find this value in the past outside the concept of ecosystem services (Bartlett et al., 1993, 2002; Van Tassell et al., 1997; Torell et al., 2003; Rimbey and Torell, 2011; Vincent, 2019). Federal forage value can be thought of as a non-market good because the federal grazing fee is set by the federal government rather than resulting from a competitive market (Quigley and Tanaka, 1988). Ranchers with public land permits or leases currently pay an annual fee per AUM to graze which is determined by the Public Rangeland Improvement Act (**PRIA**) fee formula. The PRIA fee formula includes the Beef Cattle Price Index and the Prices Paid Index (Vincent, 2019). This fee has been argued to be purposefully low in order to account for ranchers’ “ability to pay” rather than to capture the fair market value of forage or to recoup agencies expenditures (GAO, 2005).

Because of the complex history and nature of assigning values to forage on federal lands, several methods were considered by the authors. The methods reviewed included 1) modeling the value of an AUM lost using linear programing (**LP**) models of representative cow–calf public land ranches in Idaho, Oregon, Nevada, Wyoming developed from 2017 enterprise budgets (Hilken et al., 2018), 2) the recommendations from the study by Bartlett et al. (1993), and 3) 2017 USDA NASS survey indications of monthly lease rates for private, nonirrigated grazing land for the 16 states governed by the grazing fee (NASS, 2019). LP modeling resulted in an estimated average value of $24.00/AUM compared with $22.60/AUM as estimated from the NASS private lease rates. The range suggested by the study by Bartlett et al. (1993) was updated to 2017 using the Forage Value Index (per recommendation by that study), which gave an estimated range of $16.27/AUM to $27.12/AUM.

While the LP and the NASS private lease rate estimates were within the recommended range from Bartlett et al., 1993), this study used the NASS private lease rate for each U.S. state (NASS, 2019), as it was the most readily available and geographically specific option. The value ($/AUM) was then multiplied by the number of AUMs billed (in the case of BLM) or authorized (in the case of USFS) as given in publicly available annual reports (BLM, 2019; USFS, 2017) to get a total dollar value per state. That total value was then divided by the area grazed by cattle in each U.S. state to arrive at a U.S. state-specific per hectare value for forage on federal lands.

### Other Ecosystem Services

The value of other ecosystem services was estimated using CRP Grasslands annual rental payments as a proxy for nonspecified services (FSA, 2018). In this voluntary, federal government program, operators are allowed to graze while receiving financial payments and optional cost-share assistance to maintain animal and plant diversity. These rental payments are only eligible in practice for private lands, but in this study, this value is considered to be applicable to both federal and private grasslands as the best available, geographically specific, monetary estimate of ecosystem services other than from recreation and forage, for example, biodiversity.

## RESULTS AND DISCUSSION

### Beef Cattle Ranching- and Farming-Based Ecosystem Services—United States

**Figure 1.**
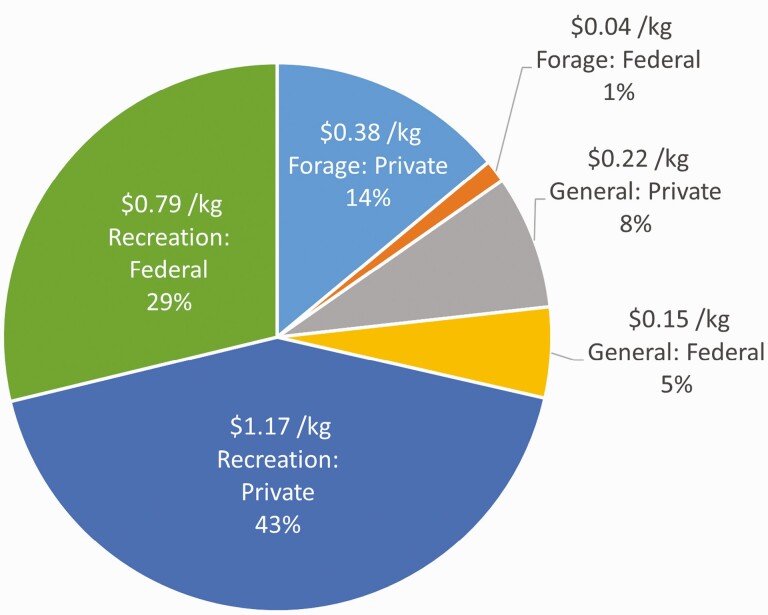
The breakdown of ecosystem service benefits from beef cattle ranches and farms as attributed to each of the three categories (recreation, forage, and other) considered for this study and according to the contribution from federal versus private rangelands and pastures. Results are shown in U.S. dollars per kilogram of retail beef and as percent of the total value ($24.5 billion).

The estimated economic value of ecosystem services from beef cattle ranches and farms was $17.5 billion for wildlife recreation, $3.8 billion for forage production, and $3.2 billion for other ecosystem services. The combined total of these estimates was $24.5 billion, of which 35% originated from federal rangelands and 65% from private rangelands and pasture. This value also represents $1,043.35 of ecosystem services per beef cow and $2.74 of ecosystem services per kilogram of retail beef. Additional details about these results, including ecosystem service values for each U.S. state can be found in Maher et al. (2020). Figure 1 shows the per kilogram estimated ecosystem services value for the United States in 2017 for each ecosystem service category considered. In line with Rashford et al. (2013) and Taylor et al. (2019), this study finds that policy or management analysis that overlooks ecosystem service flows from wildlife recreation and other ecosystem services will considerably underestimate the benefits humans derive from ecosystem services supported by cattle ranches and farms. Rashford et al. (2013) advocated for the inclusion of ecosystem services values from cattle production above that of just forage production in benefit-cost analysis and public policy considerations.

These total values are 65% higher than recent estimates by Taylor et al. (2019). There are two reasons for this difference. The first reason is that Taylor et al. (2019) did not consider ecosystem services from federal grazing lands, which highlights the importance of including them in such valuations. The second reason is that private rangelands and pastures as reported in the 2012 Census of Agriculture were 6% lower than those reported in the 2017 Census of Agriculture. The disparity in production levels may be attributable to economic factors and widespread drought in 2012, indicating that these values as calculated can fluctuate depending on such conditions.

### The Distribution of Ecosystem Services in the United States

**Figure 2.**
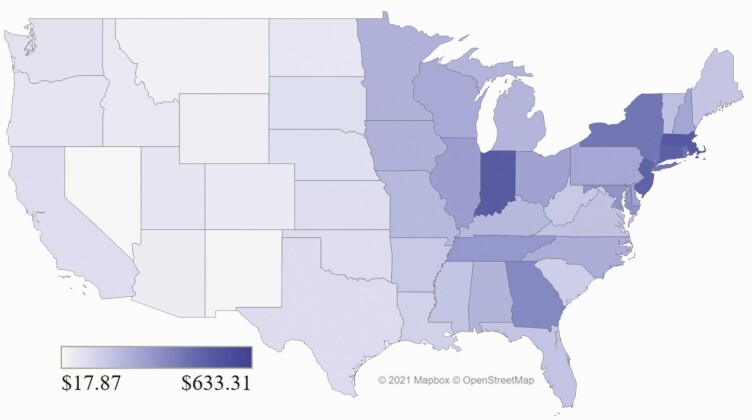
Weighted average U.S. dollar value of ecosystems services per hectare in each U.S. state, determined as the sum of the federal per hectare value multiplied by the percentage of total hectares from federal land and the private per hectare value multiplied by the percentage of total hectares from private land.

Figure 2 illustrates the dollar value of ecosystems services per hectare for each U.S. state as an area-weighted average of federal and private rangeland. Eastern states tend to have the highest ecosystem service values per hectare because they have relatively large population bases, relatively small land areas, and no federal land. As a result, the number of recreation days per unit land area tended to be relatively higher which increased the ecosystem service values per hectare. Recreation days per hectare explained 90% of the variation in the calculated value of recreation per hectare. Nationally, the range of number of recreation days per unit area was wide: Connecticut had 11.8 recreation days per hectare, whereas Nevada had 0.2 recreation days per hectare. The other ecosystem service category of value and forage production values were also higher in eastern states.

**Figure 3.**
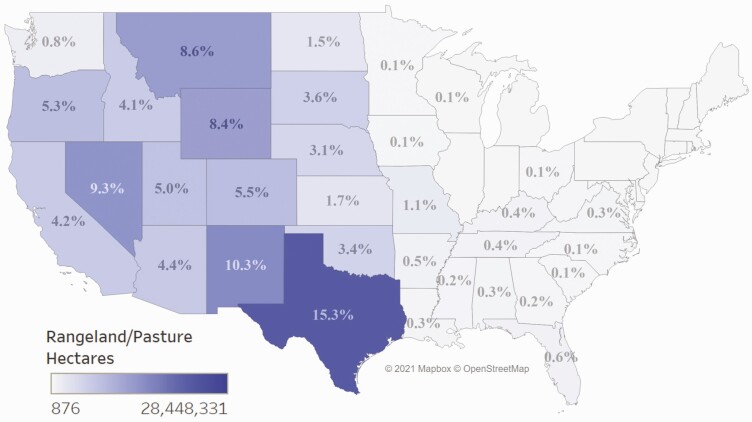
Total federal and private rangeland/pasture hectares estimated to be grazed by cattle in each state in 2017 and those hectares as a percentage of the total hectares calculated for the continental U.S. U.S. States with less than 0.05% of the total hectares are not labeled.

**Figure 4.**
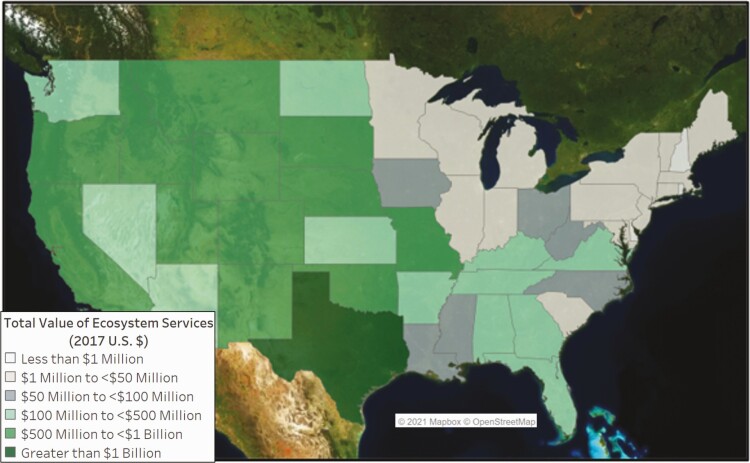
Cattle ranching-based ecosystem services value by U.S. state.

Although there is a high value per hectare in some of the eastern states, this study found that total ecosystem services values (value per hectare multiplied by total hectares in that state) provided the best representation of the geographic distribution of these ecosystem services across the United States. Figure 3 depicts the distribution of beef cattle ranching rangeland/pasture hectares across the country and for each state as a percent of the total number in the continental United States—for example, 15.3% of U.S. hectares used for cattle ranching are in Texas. The majority of cattle ranches and farm rangelands/pastures hectares are located in the western half of the Unites States. The 17 U.S. states from the Great Plains Region and westward contain nearly 95% of the rangeland/pastures used for beef cattle grazing in the United States. Figure 4 provides the total ecosystem service values for each state. Approximately 52% of the states considered in this study had an ecosystem services value from beef cattle ranches and farms of over $100 million. States with estimated values of $500 million or more were primarily from the Great Plains westward.

**Figure 5.**
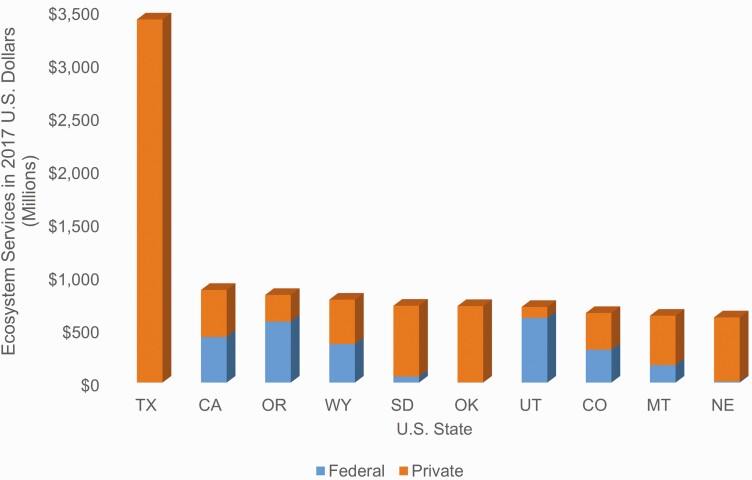
Top 10 U.S. states in terms of total value of cattle ranching-based ecosystems services shown according to the relative contribution from federal vs. private land.

The top 10 U.S. states in terms of total value from ecosystem services are shown in Figure 5. Seven of the top 10 states are part of the Great Plains region. Three states (California, Oregon, and Utah) in the top 10 had nearly 50% or more of their value in ecosystem services coming from federal rangelands. Total ecosystem service values were generally higher in the western states, despite lower per hectare values, than in the east. The variation in total value is driven mostly by variation in hectares. The total number of rangeland/pasture hectares in the state was not the only factor, however. Per hectare values also matter. For example, Wyoming had a higher percentage (8.4%) of total calculated beef cattle ranching rangeland/pasture hectares than California (4.2%; Figure 3), yet the estimated per hectare values of ecosystem services on private land and federal land in Wyoming were both less than half that found for California. As a result, Wyoming had a lower total value of ecosystem services as compared to California ($782 million vs. $873 million) even though the state had more rangeland/pasture hectares. Texas stands apart from the other states in the United States, with more than 28.3 million (15.3%) rangeland/pastures hectares used for beef cattle ranching and all of the estimated ecosystem service value in the state coming from private land. This state produced over 1/5 of the total U.S. ecosystem service values (as summed over each individual state) from beef cattle ranches and farms. These ranches and farms in Texas produced nearly 4 million head of beef cows (17% of industry production) in 2017 (USDA, 2019), making it the largest cattle ranching state in the nation, by far.

### Opportunities and Challenges

**Figure 6.**
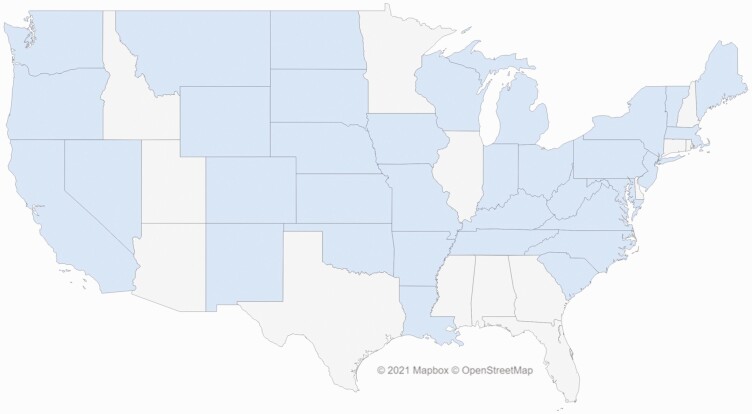
U.S. states that showed a decrease in the value of ecosystem services per beef cow of 5% or more (shaded area), comparing results given data from the 2017 versus 2012 Census of Agriculture.

There are several opportunities and challenges associated with the valuation and incorporation of cattle ranching-based ecosystem services into existing decision-frameworks. One challenge is the temporal fluctuations in values. The values reported in this study correspond to a point in time, but the total and per cow ecosystem services values vary from year to year as rangeland/pasture hectares utilized and beef cow numbers fluctuate. The percent change in ecosystem services value per beef cow declined for most states when using data from the Census of Agriculture in 2017 when compared with 2012. Changes ranged from a decrease of 30% to an increase of 29%. Thirty-four states saw a decline in value of 5% or more (Figure 6). The decline in ecosystem services value per beef cow is the result of more beef cows in production in 2017 when compared with 2012. The temporal scope is therefore an important consideration that can be limited by available data. The values from federal land can also fluctuate over time though these differences are not examined here due to data limitations; GIS data used in this analysis to determine federal land area is dated to 2017 and there was no way that the authors could find to draw information from 2012 specifically.

It should also be noted that this study is not meant to be a net accounting of the value to society from cattle production. There are additional economic values associated with cattle ranching that have not been incorporated here. For example, the land, buildings, machinery, and equipment associated with this industry was estimated to be $655.4 billion (up 25% from that reported in the 2012) and the industry employed over 2.1 million (up 10% from that reported in the 2012) workers including operators, hired labor, and family labor in 2017. Also, there are ecosystem service costs associated with beef cattle ranching (Pogue et al., 2018; Rotz et al., 2019). A net accounting requires further analysis. At the management level, evaluating trade-offs and synergies between different ecosystem services (e.g., the relationship between changes in forage provisioning services and erosion control) across space and time is an important next step possibly requiring experiment-based mechanistic studies that have been argued to be lacking in number (Zhao et al., 2020).

This study provides lower bound estimates of the value of all the ecosystem services provided by land used in beef cattle production for two reasons. First, these estimates may not capture important ecosystem services that are more difficult to quantify and value, although some of these may be captured by using the CRP Grasslands payments as an approximation of other ecosystem services. Some of the ecosystem services that may be omitted or undervalued here include the supply of water, being part of alternative energy production such as wind or solar (Brunson et al., 2016), sustaining biodiversity (Havstad et al., 2007), sequestering carbon (Havstad et al., 2007; Rashford et al., 2013), and providing cultural benefits, including protection of a way of life (Gentner and Tanaka, 2002; Lind, 2015; Collins, 2019). Second, due to data limitations, beef production from other industry classifications other than NAICS 112111 was not included. In 2017, other types of agricultural operations produced 32% of agricultural beef cattle in the United States but had most of their operation in other types of livestock or production activities. For example, the sheep and goat farming industry classification reported 4.6 million hectares in rangeland/pasture in 2017. Although this industry produced 98,000 beef cows, it also showed a total inventory of 3.4 million sheep and lambs and 1.6 million goats. Assigning the hectares from rangelands/pastures to these different livestock types is not possible from the data in the Census alone and would require additional assumptions.

The estimated dollar values per hectare provided by this study can be applied in impact analysis or for planning purposes with caution. Ecosystem services values per unit area for each U.S. state can be found in Maher et al. (2020). Important considerations for such applications include 1) possible finer scale variation in value at the project-level than in our state-level estimates, 2) understanding impact functions that may be nonlinear and discontinuous, and 3) the potential for synergistic relationships between private and federal ecosystem services changes. The latter two may be easily overlooked because they are unique to ranching. Finer scale variation in per area values refers to the idea that ecosystem services vary across space. The actual variability in the values estimated may be greater when considering finer spatial resolutions than what is represented by the state-level averages reported here.

Nonlinear and potentially discontinuous impact functions mean that each additional hectare of rangeland/pasture transferred out of ranching and into other land uses does not have a constant effect. The impacts per unit area on operators and/or ecosystem services may increase at an increasing rate and at some points may experience large discontinuous jumps in impact. For example, ranchers may go out of business even when a part of their forage base is affected (Maher et al., 2013; Torell et al. 2014a; Runge et al. 2019). This may make an operator more likely to convert private rangelands or pastures to cropping, or sell them, possibly for development. Land use change in one area could also affect the set of ecosystem services from nearby lands, for example, declines in the availability of goods and services needed for ranching in an area, disruption of wildlife habitat corridors, increases in stormwater run-off, and/or affect other economic and environmental synergies tied to the existing land use. In summary, there is potential for the project impact to go beyond the boundaries of the project itself.

Ecosystem service values from private and public land could be affected by one another as well, resulting in synergistic effects. However, the estimates presented here are calculated additively. Private and federal grazing use are interrelated because cattle that graze on federal land also graze on private land for part of the year. The result of these synergistic effects would depend on the situation. A decline in the availability of grazing on federal lands could affect demand for private land which would be reflected in higher rental rates. In the West, however, private land has been in short supply or unaffordable leaving few alternatives to federal land forage. Therefore, transportation costs and other factors that affect profit margins can make it more practical to reduce herd sizes or get out of ranching altogether (Torell et al., 2014a). A number of studies have explored the possible unintended consequences of declines in federal grazing land availability (e.g., grazing restrictions) and subsequent declines in beef production (Torell et al., 2014a, b; Runge et al. 2019; Lewin et al., 2019). Such changes in federal land availability could have unforeseen environmental consequences by making development investment opportunities on private land more attractive to private landowners (Runge et al., 2019). Synergistic relationships between private and federal ecosystem service values are an important area for future research.

### Policy, Management, and Planning Applications

Conserving rangeland ecosystem services through preserving working rangelands has been the focus of conservationists in the United States for more than two decades (Maestas et al., 2002; Havstad et al., 2007; Brunson and Huntsinger, 2008; Maczko et al., 2011) and remains a societal concern as evidenced in several recent studies (Hongli et al., 2013; Allred et al., 2015; Lark et al. 2015; Haggerty et al., 2018; WWF, 2018; Runge et al., 2019). Helping ranchers stay afloat as competition from other land uses increase can be supported through managing incentives (Havstad et al., 2007; Bryan et al., 2013; Farley et al., 2017) and working groups, such as the Sustainable Rangelands Roundtable (Maczko et al., 2016). Among other support mechanisms, this group developed tools that can weigh the benefits and costs of generating income from less traditional ecosystem services (Maczko et al., 2011; Maczko et al., 2016). Establishing sound methods to incorporate the value of these less traditional ecosystem services into policy and planning is also critical.

**Figure 7.**
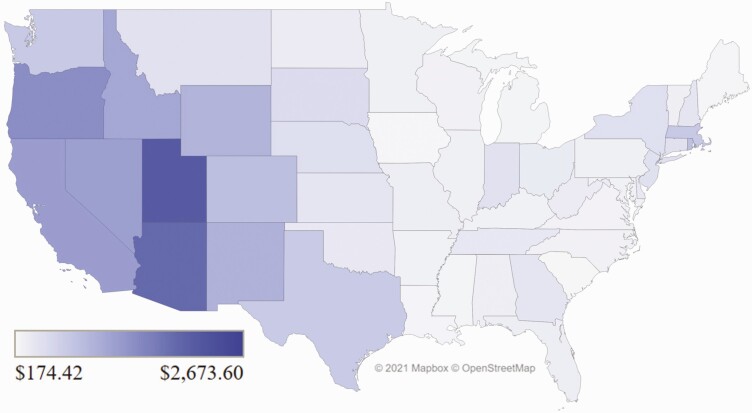
Beef cattle ranching-based ecosystem services value per beef cow.

**Figure 8.**
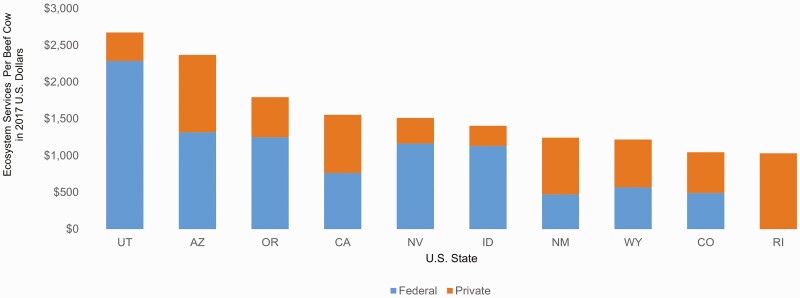
States in the top ten for cattle ranching-based ecosystem services values per beef cow, broken down according to the relative contribution from federal versus private land.

The main production unit from beef cattle ranching is the quantity of beef cows produced, and therefore sales value may seem like an obvious choice for estimating the societal value of this production. However, this measurement may undervalue the societal contribution of cattle production in certain areas. Figure 7 shows the distribution of the estimated ecosystem services value on a per cow basis. Figure 8 provides the states in the top 10 of value per beef cow. Hectares per beef cow in the 48 contiguous U.S. states ranged from 84.6 in Nevada to 0.7 in Maryland. Utah and Arizona (both in the top five of rangeland/pasture area per beef cow) were found to have the highest value of ecosystem services per beef cow (Figures 7 and 8). However, both of these states were ranked lower than other states in sales value per beef cow (44th and 37th, respectively). Sales values per beef cow was found by dividing the value from cattle and calves sales in each state by the total number of beef cows produced by beef cattle ranches and farms (NASS, 2017). In 2017, the sales value per beef cow in Utah was $1,007 (vs. $2,674 in ecosystem services) and $757 (vs. $2,367 in ecosystem services) per beef cow in Arizona.

**Figure 9.**
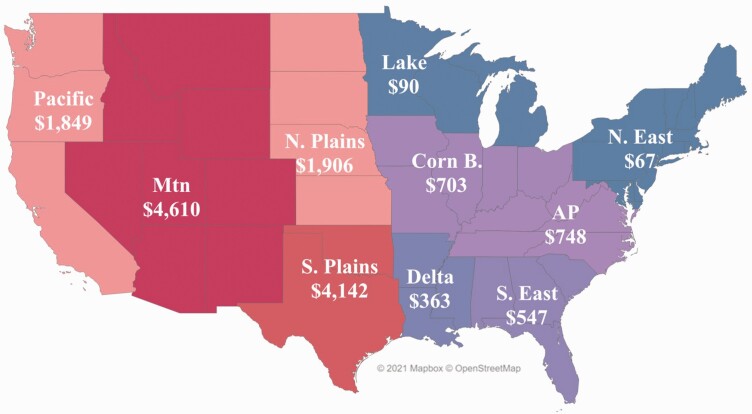
The distribution of estimated cattle ranching-based ecosystem services (millions of U.S. dollars) in 2017 grouped by economic regions in USDA NASS Land Values Summary, 2018 (NASS, 2018).

Another example of the importance of considering values other than direct income generation in policy and planning can be seen by comparing the geographic distribution of total cattle ranching-based ecosystem services (Figure 9) versus the distribution of cattle/calves sales (Table 2) using NASS-defined economic regions. In 2017, the Plains (north and south), Mountain, and Pacific regions provided 83% of the total national value of ecosystem services from cattle ranching and 69% of the total value of cattle/calves sales. Comparing regions in the West, the combined sales value in the S. and N. Plains ($13.3 billion) is almost twice that of the Pacific and Mountain regions ($6.9 billion), yet the combined ecosystem services value is approximately 7% more in the Pacific and Mountain regions than in the Plains regions. In addition, the calculated percentage of ecosystem service value from federal rangeland in the Pacific and Mountain regions is 58% and 56%, respectively. This suggests that public land management and policy can greatly influence the value of ecosystem services in these areas.

**Table 2.** Cattle and calves sales in 2017 grouped by economic region in USDA NASS Land Values Summary, 2018 (NASS, 2018)

Region	U.S. states	Cattle/calves sales (millions of U.S. dollars)
West		
Southern Plains	OK, TX	$6,754
Northern Plains	KS, ND, NE, SD	$6,620
Mountain	AZ, CO, ID, MT, NM, NV, UT, WY	$4,928
Pacific	CA, OR, WA	$1,981
Total		$20,284
East		
Corn Belt	IA, IL, IN, MO, OH	$2,925
Appalachian	KY, NC, TN, VA, WV	$2,297
Lake States	MI, MN, WI	$1,255
Delta States	AR, LA, MS	$1,179
Southeast	AL, FL, GA, SC	$1,067
Northeast	CT, DE, MA, MD, ME, NH, NJ, NY, PA, RI, VT	$381
Total		$9,105

**Figure 10.**
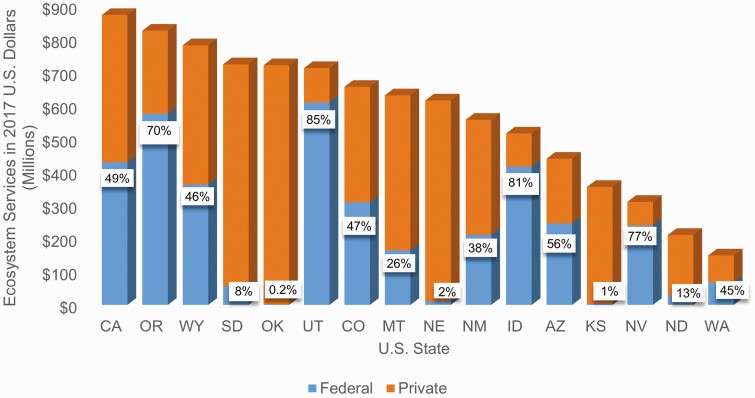
Total cattle ranching-based ecosystem services for the U.S. states that have federal grazing land and percentage of that total value that is from federal grazing land.

Future research could consider how different options in land use and management affect the suite of ecosystem services from rangelands. This is especially important on federal grazing lands where there is a significant research gap in the understanding of land management options, land use change, and their impact on ecosystem services (Torell et al., 2014c). In some areas, cattle ranches and farms may rely heavily on federal land management for their operation and ecosystem services in these areas may be affected disproportionately by federal policy and land management decisions as compared to other areas. For example, the states of Utah and Oklahoma (Figure 10) were found to have similar total values in cattle ranching-based ecosystem services, yet 85% of this value in the state of Utah was from federal grazing lands whereas less than 0.5% of this value was from federal land in Oklahoma.

This study provided a systematic look into the potential for valuing ecosystem services associated with beef cattle ranching and the rangelands and pastures that support them. While concern for ecosystem services has increased since the Millennium Ecosystem Assessment (MEA, 2005), it is difficult to ensure their consideration and protection in policy and management. More recently, there has been a drive to incorporate ecosystem services into decision-making at the federal agency level (Donovan et al., 2015; NESP, 2016; Deal et al., 2017; Olander et al., 2018). Studies like the one presented here are needed to inform decision-makers and may help to conserve ecosystem service flows from rangelands and pastures moving into the future.
